# Effect of Short-Chain Fatty Acids on Inflammatory and Metabolic Function in an Obese Skeletal Muscle Cell Culture Model

**DOI:** 10.3390/nu16040500

**Published:** 2024-02-09

**Authors:** Kelsey Van, Jessie L. Burns, Jennifer M. Monk

**Affiliations:** 1 Department of Human Health and Nutritional Sciences, University of Guelph, Guelph, ON N1G 2W1, Canada; kvan@uoguelph.ca; 2 Department of Health Sciences, Carleton University, Ottawa, ON K1S 5B6, Canada; jessie.burns@carleton.ca

**Keywords:** obesity, L6 myotubes, skeletal muscle, short-chain fatty acids, inflammation, lipopolysaccharide, palmitic acid, cytokines, chemokines, myokines, insulin-stimulated glucose uptake

## Abstract

The fermentation of non-digestible carbohydrates produces short-chain fatty acids (SCFAs), which have been shown to impact both skeletal muscle metabolic and inflammatory function; however, their effects within the obese skeletal muscle microenvironment are unknown. In this study, we developed a skeletal muscle in vitro model to mimic the critical features of the obese skeletal muscle microenvironment using L6 myotubes co-treated with 10 ng/mL lipopolysaccharide (LPS) and 500 µM palmitic acid (PA) for 24 h ± individual SCFAs, namely acetate, propionate and butyrate at 0.5 mM and 2.5 mM. At the lower SCFA concentration (0.5 mM), all three SCFA reduced the secreted protein level of RANTES, and only butyrate reduced IL-6 protein secretion and the intracellular protein levels of activated (i.e., ratio of phosphorylated–total) NFκB p65 and STAT3 (*p* < 0.05). Conversely, at the higher SCFA concentration (2.5 mM), individual SCFAs exerted different effects on inflammatory mediator secretion. Specifically, butyrate reduced IL-6, MCP-1 and RANTES secretion, propionate reduced IL-6 and RANTES, and acetate only reduced RANTES secretion (*p* < 0.05). All three SCFAs reduced intracellular protein levels of activated NFκB p65 and STAT3 (*p* < 0.05). Importantly, only the 2.5 mM SCFA concentration resulted in all three SCFAs increasing insulin-stimulated glucose uptake compared to control L6 myotube cultures (*p* < 0.05). Therefore, SCFAs exert differential effects on inflammatory mediator secretion in a cell culture model, recapitulating the obese skeletal muscle microenvironment; however, all three SCFAs exerted a beneficial metabolic effect only at a higher concentration via increasing insulin-stimulated glucose uptake, collectively exerting differing degrees of a beneficial effect on obesity-associated skeletal muscle dysfunction.

## 1. Introduction

It is estimated that globally over 600 million adults are obese [1]. Obesity is associated with metabolic cardiovascular risk factors, including but not limited to dyslipidemia, insulin resistance and hypertension, which increases the likelihood of developing co-morbidities such as cardiovascular disease and type 2 diabetes [2]. The gastrointestinal microenvironment (microbiome and host mucosa) is also altered in obesity, wherein the composition of the microbiome differs between obese and non-obese states and epithelial barrier permeability increases, leading to the translocation of bacterial endotoxins into the systemic circulation and subsequent increases in inflammation and metabolic dysfunction [3,4,5]. The increased dietary intake of non-digestible carbohydrates (NDCs) can be fermented by the microbiome and has been shown to improve both intestinal and extra-intestinal elements of the obese phenotype, effects that are largely attributed to the fermentation products’ short-chain fatty acids (SCFAs) [6,7,8,9,10], as reviewed in [4]. 

The production of SCFAs will differ between individuals based on the dietary intake of fermentable precursors (predominantly NDC and to a lesser degree from undigested proteins) and both the activity and composition of the microbiome (namely the relative abundance of SCFA-producing versus non-SCFA-producing species) [11,12,13,14]. Consequently, there is considerable variability in the reported fecal SCFA levels in humans, ranging from 20 to 150 mM, produced in a typical 3:1:1 molar ratio of acetate–propionate–butyrate, respectively [15,16,17]. Although SCFAs can impact the gastrointestinal microenvironment they are produced within [3,5], they also reach systemic circulation at lower concentrations compared to fecal levels [18,19,20,21,22,23] and can impact metabolic function extra-intestinally, such as in skeletal muscles, adipose tissue, liver, and pancreas [24,25,26,27]. Specifically, within skeletal muscle, the metabolic effects of SCFAs have been focused on acetate, with few studies investigating the potential effects of propionate or butyrate [28,29,30,31,32,33,34,35]. Within skeletal muscle or skeletal muscle cell lines, acetate has been shown to increase (i) glucose tolerance and/or insulin-stimulated glucose uptake [36], (ii) skeletal muscle mRNA expression of glucose transporter type 4 (GLUT4) [28,29], and (iii) glucose uptake [30]. Similarly, propionate has been shown to increase glucose uptake [30], whereas butyrate did not [31]; however, insulin signaling was improved by butyrate following palmitic acid (PA)-induced insulin resistance [31]. Furthermore, a high-fat diet combined with sodium butyrate supplementation (5% *w*/*w*) reduced homeostasis model assessment-estimated insulin resistance (*HOMA*-*IR*), fasting blood glucose, and insulin levels [28,32]. Collectively, these data indicate that individual SCFAs can exert different effects on critical aspects of skeletal muscle function. 

Proinflammatory mediators (cytokines and chemokines), such as tumor necrosis factor (TNF)α; interleukin (IL)-6; monocyte chemoattractant protein (MCP)-1; regulated on activation, normal T cell expressed and secreted (RANTES); and macrophage inflammatory protein (MIP)-1α (amongst others), can adversely impact skeletal muscle function in obesity and/or recruit immune cells in obese and insulin-resistant skeletal muscle and promote pro-inflammatory cross-talk between immune and skeletal muscle cells [37,38,39]. Previously, we have shown that inflammatory transcription factor activation (phosphorylated-NFκB p65) levels and the secretion of inflammatory cytokines and chemokines (i.e., myokines), including MCP-1 and MIP-1α RANTES, are reduced by a low dose (0.5 mM) of butyrate in response to LPS stimulation, whereas acetate and propionate had no effect [40]. This indicated that not all SCFAs exert the same biological effects and that these effects are concentration-dependent. This was underscored by the finding that a higher SCFA (2.5 mM) concentration combined with an inflammatory stimulus via lipopolysaccharide (LPS) resulted in butyrate exhibiting a potent non-inflammatory effect, reducing the activation/phosphorylation of both NFκB p65 and STAT3 transcription factors and the secretion of downstream myokines, including IL-6, MCP-1, MIP-1α, and RANTES, whereas propionate had an intermediate effect when reducing the activation of NFκB p65 and the secretion of MCP-1, and RANTES and acetate had no effect on myokine secretion but reduced the activation of NFκB p65 [40]. Similarly, in response to palmitic acid (PA) stimulation, which induces insulin resistance [41,42], L6 myotubes treated with butyrate at both a low and high concentration (0.5 mM and 2.5 mM, respectively) had a potent inhibitory effect on inflammatory transcription factor activation (NFκB p65 and STAT3) and myokine secretion (IL-6, MCP-1, MIP-1α, and RANTES) [40]. Conversely, following PA stimulation, 0.5 mM propionate and acetate modestly affected myokine secretion; however, at 2.5 mM concentration, both propionate and acetate reduced transcription factor activation and the secretion of the same inflammatory myokines that are also reduced by butyrate, and their effect was less potent compared to butyrate. Although both LPS and PA stimulation conditions individually recapitulate critical aspects of the obese skeletal muscle phenotype, their combined effects more accurately reflect the critical features of the obese skeletal muscle cellular microenvironment of combined endotoxin-induced inflammation and insulin resistance [37,38,41,42,43,44,45,46]. 

Thus, the objective of the current study was to determine the effects of individual SCFAs (namely acetate, propionate, and butyrate) on combined LPS + PA-stimulated L6-myotube-derived inflammatory mediator secretion and the subsequent impact on metabolic function in a skeletal muscle cell culture model that reflects the obese skeletal muscle’s cellular microenvironment.

## 2. Materials and Methods

### 2.1. L6 Myoblast Cell Culture Conditions and Differentiation

L6 myoblasts were purchased from the American Type Culture Collection (ATCC; CRL-1458; Manassas, VA, USA), and they were grown and passaged according to the manufacturer’s specifications and as described previously [40]. In brief, cells were maintained in a humidified incubator at 5% carbon dioxide and 37 °C, in Dulbecco’s modified Eagle’s medium (DMEM) containing 4 mmol/L L-glutamine, 4500 mg/L glucose, and 1 mM sodium pyruvate (HyClone, Logan, UT, USA), which was supplemented with 10% (*v*/*v*) low endotoxin sterile-filtered fetal bovine serum (FBS; Millipore-Sigma, Oakville, ON, Canada) and 1% (*v*/*v*) penicillin-streptomycin (HyClone) [30,31,47]. Myoblasts were seeded at 4 × 10^4^ cells/mL in 6-well plates (Fisher Scientific, Mississauga, ON, Canada), and upon reaching 70–80% confluence, they were differentiated and maintained as multi-nucleated myotubes with striated fibers in DMEM supplemented with 2% (*v*/*v*) FBS and 1% (*v*/*v*) penicillin-streptomycin. Media were changed on days 2, 4, and 6, and on day 7, the media were replaced with serum-reduced DMEM containing 0.25% (*v*/*v*) FBS and 1% (*v*/*v*) penicillin-streptomycin for 12 h prior to experiments on day 8.

### 2.2. L6 Myotube Experimental Treatment Conditions

L6 myotube cultures were treated with a combined stimulus containing 10 ng/mL of LPS from *Escherichia coli* 055:B5 (Millipore-Sigma) plus 500 µM of PA (≥98% pure, Caymen Chemicals, Ann Arbor, MI, USA) to mimic an obese skeletal muscle cellular microenvironment. The dose of LPS recapitulates the circulating endotoxin levels reported in obese humans [45] and high-fat-diet-induced obesity rodent models [9,46]. Furthermore, the dose of PA has previously been shown to induce insulin resistance while maintaining cell viability [41,42,43]. To generate a stock solution of PA, the fatty acid was complexed to 2% (*w*/*v*) bovine serum albumin (BSA; containing ≤0.1 ng/mg endotoxin and ≤0.02% fatty acids; Millipore-Sigma) using lab-grade ethanol and subsequently dissolved in serum-reduced DMEM pre-warmed to 37 °C prior to its addition to the L6 myotube cultures. Thus, LPS + PA-stimulated L6 myotube cultures were further stimulated ± individual SCFAs (n = 9/treatment condition). SCFA-free cultures containing media served as controls (CONs). The individual SCFAs used in the experiments were sodium acetate (ACE), sodium propionate (PRO), and sodium butyrate (BUT) (Millipore-Sigma). SCFA treatments consisted of a low concentration of 0.5 mM or a high concentration of 2.5 mM for 24 h, as used previously [28,40,48], and they reflect human circulating SCFA concentrations [18,19,20,21,22,23]. Trypan blue staining was used to assess cell viability and exceeded >95% in all experimental conditions. 

### 2.3. Secreted Protein Analysis in L6 Myotube Culture Supernatant

Secreted proteins, namely cytokines and chemokines in LPS + PA-stimulated ± SCFA-treated L6 myotube culture supernatants, were measured using a Bio-Plex Pro Cytokine 5-plex kit as per the manufacturer’s instructions. The secreted protein endpoints were selected based on previous work utilizing individual LPS- and PA-stimulated L6 myotube cultures [40] and included TNFα, IL-6, MCP-1, MIP-1α, and RANTES. Secreted protein endpoints were analyzed using the Bio-Plex 200 system and Bio-Plex Manager software, Version 6.0 (Bio-Rad, Hercules, CA, USA).

### 2.4. Inflammatory Transcription Factor Activation

Intracellular protein levels in LPS + PA-stimulated cultures were quantified using the bicinchoninic assay (Fisher Scientific, Waltham, MA, USA) according to the manufacturer’s instructions. The inflammatory transcription factor activation status was determined by separately measuring the intracellular expression level of both total and phosphorylated NFκB p65 and total and phosphorylated STAT3 using the InstantOne ELISA kits (Applied Biosystems, Waltham, MA, USA) according to the manufacturer’s instructions. The activated forms of each transcription factor were phosphorylated NFκB p65 (Ser 536) and phosphorylated STAT3 (Tyr 705). In brief, 10 μg of intracellular protein/sample was used in each of the four ELISA assays. Only LPS + PA-treated samples ± SCFAs were measured, and final absorbance was read at 450 nm (PowerWave XS2, BioTek, Winnooski, VT, USA). The activation status of each transcription factor was presented as the ratio of phosphorylated (NFκB p65 or STAT3) to total expression level.

### 2.5. Glucose Uptake Assay

Glucose uptake in L6 myotube cultures was assessed using a colorimetric Glucose Uptake Assay Kit (Abcam, ab136955, Boston, MA, USA) according to the manufacturer’s instructions. In brief, L6 myotubes were cultured, differentiated, and treated (with combined LPS + PA stimulation ± SCFA at 0.5 mM and 2.5 mM concentrations in serum-reduced DMEM), as described above in 96-well plates (2600 cells/well). After 24 h incubation with the experimental treatment conditions, L6 myotubes were washed three times with PBS prior to pre-incubating the cells with 100 μL Krebs-Ringer-Phosphate-Hepes (KRPH) buffer containing 2% BSA for 40 min at 37 °C to carry out glucose starvation with respect to the cells. Subsequently, cells were treated with ±1 µM insulin (Millipore-Sigma, Burlington, MA, USA) for 20 min, followed by the addition of 10 mM of the glucose analog, 2-deoxy glucose (2-DG), for 20 min at 37 °C. Using the extraction buffer provided with the kit, cells were lysed by pipetting up and down, and the resultant lysates were collected and snap-frozen in liquid nitrogen prior to the remaining reaction steps in the kit instructions for the treatment of cell lysates, wherein 2-DG is converted to NADPH, leading to the production of an oxidized product that was detected at an optical density (OD) of 412 nm using a spectrophotometer (Molecular Devices, San Jose, CA, USA). As observed previously [49], glucose uptake in non-insulin-stimulated L6 myotube cultures was significantly lower compared to insulin-stimulated L6 myotube cultures and there was no difference in glucose uptake in non-insulin-stimulated L6 myotube cultures ± SCFAs. 

### 2.6. Statistical Analysis

All data are expressed as mean  ±  SEM. Secreted protein levels were analyzed via one-way ANOVA (main effect: SCFA treatment). All ANOVA analyses were followed by Tukey’s multiple comparison test for post hoc analyses between groups (*p* < 0.05). The Shapiro–Wilk test was used to test for normality. Analyses were conducted using GraphPad Prism 9.3.0 (GraphPad Software, Inc., La Jolla, CA, USA). 

## 3. Results

### 3.1. Effect of a Low (0.5 mM) SCFA Concentration on Inflammatory Cytokine and Chemokine Mediator Secretion in LPS + PA-Stimulated L6 Myotube Cultures

**Figure 1:**
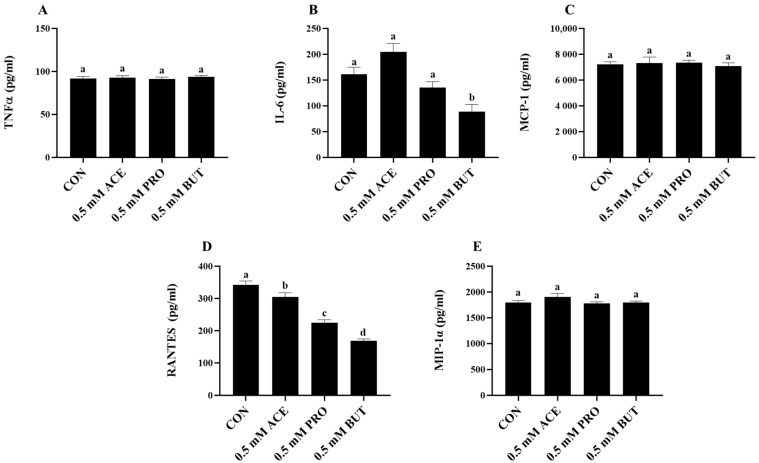
Effect of 0.5 mM SCFA + combined LPS and PA stimulation on L6 myotube-secreted protein levels (**A**–**E**). Values are means ± SEM. Data were analyzed via one-way ANOVA followed by Tukey’s multiple comparison test. Bars not sharing a lower-case letter differ (*p* < 0.05).

Inflammatory mediator secretion from both unstimulated L6 myotubes (media alone, negative control) and L6 myotube cultures treated only with each individual SCFA at the 0.5 mM concentration was below the limit of detection. Conversely, detectable levels of all secreted inflammatory mediators were observed in response to the combined LPS + PA stimulation condition ± 0.5 mM concentration of each individual SCFA, as shown in Figure 1. Only BUT reduced the level of secreted IL-6 compared to CON (*p* < 0.05; Figure 1B), whereas there was no difference in IL-6 secretion between CON-, ACE-, and PRO-treated L6 myotube cultures. Additionally, all three types of SCFAs significantly reduced RANTES secretion compared to CON, wherein BUT had the lowest secretion level, followed by PRO and then ACE (*p* < 0.05; Figure 1D). Conversely, there was no difference in TNFα, MCP-1, or MIP-1α secretion between CON and any SCFA treatment groups (ACE, PRO, or BUT) (*p* > 0.05; Figure 1A,C,E).

### 3.2. Effect of Low (0.5 mM) SCFA Concentration on Inflammatory Transcription Factor Activation Status in LPS + PA Stimulated L6 Myotube Cultures

**Figure 2:**
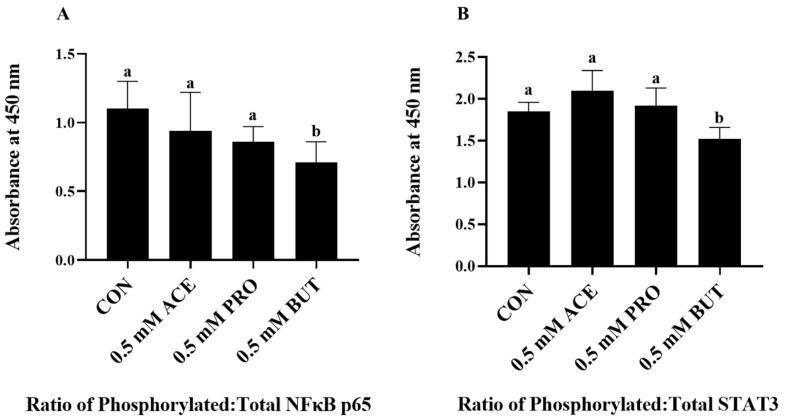
Effect of 0.5 mM SCFA + combined LPS and PA stimulation on L6 myotube intracellular protein levels of NFκB p65 and STAT3 presented as a ratio of phosphorylated–total ((**A**) and (**B**), respectively). Values are means ± SEM. Data were analyzed via one-way ANOVA, followed by Tukey’s multiple comparison test. Bars not sharing a lower-case letter differ (*p* < 0.05).

The inflammatory transcription factor activation status (namely, the ratio of phosphorylated to total intracellular protein expression) was measured in only LPS + PA-stimulated L6 myotube cultures, as shown in Figure 2. The activation of NFκB p65 was reduced by BUT compared to CON, ACE, and PRO myocyte cultures (*p* < 0.05; Figure 2A). Conversely, there was no difference in NF and NFκB p65 activation statuses between CON- and ACE- or CON- and PRO-treated myocyte cultures. Similarly, only BUT reduced the STAT3 activation status compared to CON (*p* < 0.05; Figure 2B), whereas ACE and PRO had no effect on STAT3 activation compared to CON. 

### 3.3. Effect of a Higher (2.5 mM) SCFA Concentration on Inflammatory Cytokine and Chemokine Mediator Secretion in LPS + PA-Stimulated L6 Myotube Cultures

**Figure 3:**
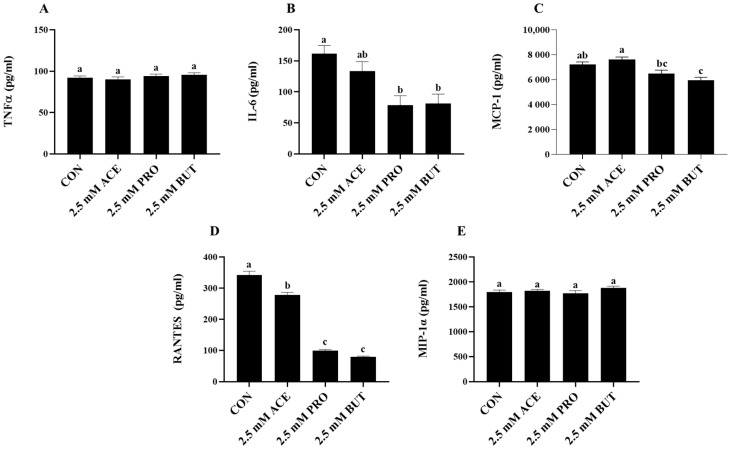
Effect of 2.5 mM SCFA + combined LPS and PA stimulation on L6 myotube-secreted protein levels (**A**–**E**). Values are means ± SEM. Data were analyzed via one-way ANOVA followed by Tukey’s multiple comparison test. Bars not sharing a lower-case letter differ (*p* < 0.05).

Inflammatory mediator secretion from unstimulated L6 myotubes (media alone, negative control) and L6 myotube cultures treated only with each individual SCFA at the 2.5 mM concentration were below the limit of detection. Therefore, the effect of individual SCFAs at the higher 2.5 mM concentration on inflammatory cytokine and chemokine secretion from LPS + PA-stimulated L6 myotubes is shown in Figure 3. Both PRO and BUT reduced IL-6 secretion compared to CON (*p* < 0.05); however, there was no effect of ACE on IL-6 secretion, which did not differ from CON (Figure 3B). Only BUT reduced MCP-1 secretion compared to CON (*p* < 0.05; Figure 3C), whereas PRO and ACE did not differ from CON. All three individual SCFAs reduced RANTES secretion compared to CON, wherein PRO and BUT had the greatest effect and did not differ from each other, and ACE exhibited an intermediate level of RANTES secretion (*p* < 0.05; Figure 3D). There was no difference in TNFα and MIP-1α secretion between CON and any SCFA treatment groups (*p* > 0.05; Figure 3A,E). 

### 3.4. Effect of a Higher (2.5 mM) SCFA Concentration on Inflammatory Transcription Factor Activation Status in LPS + PA-Stimulated L6 Myotube Cultures

**Figure 4:**
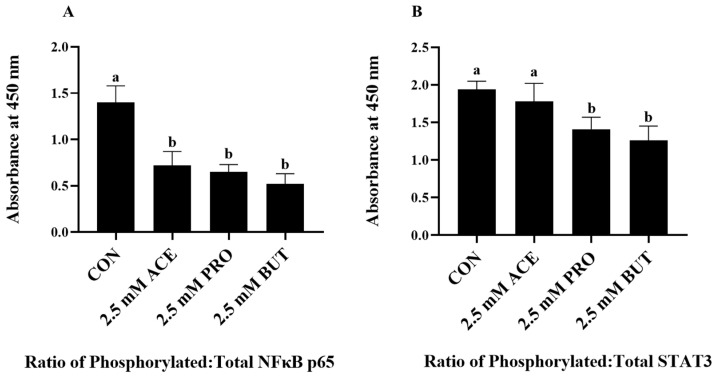
Effect of 2.5 mM SCFA + combined LPS and PA stimulation on L6 myotube intracellular protein levels of NFκB p65 and STAT3 presented as the ratio of phosphorylated–total ((**A**) and (**B**), respectively). Values are means ± SEM. Data were analyzed via one-way ANOVA followed by Tukey’s multiple comparison test. Bars not sharing a lower-case letter differ (*p* < 0.05).

The effect of a higher concentration (2.5 mM) on the ratio of phosphorylated to total intracellular protein expression of two critical inflammatory transcription factors following the LPS + PA stimulation of L6 myotube cultures is shown in Figure 4. The activation status of NFκB p65 was reduced by all SCFAs compared to CON (*p* < 0.05; Figure 4A); however, there was no difference in the NFκB p65 activation status observed between ACE-, PRO-, and BUT-treated L6 myotube cultures. The ratio of phosphorylated to total STAT3 expression was reduced in both PRO- and BUT-treated L6 myotube cultures compared to CON (*p* < 0.05; Figure 4B); however, their expression level did not differ from each other. In contrast, ACE had no effect on the STAT3 activation status, as the expression level did not differ between ACE- and CON-treated cultures (*p* > 0.05) or ACE- and either PRO- or BUT-treated cultures (*p* > 0.05). 

### 3.5. Effect of SCFA (at 0.5 mM and 2.5 mM Concentrations) on Insulin-Stimulated Glucose Uptake in LPS + PA-Stimulated L6 Myotube Cultures

**Figure 5:**
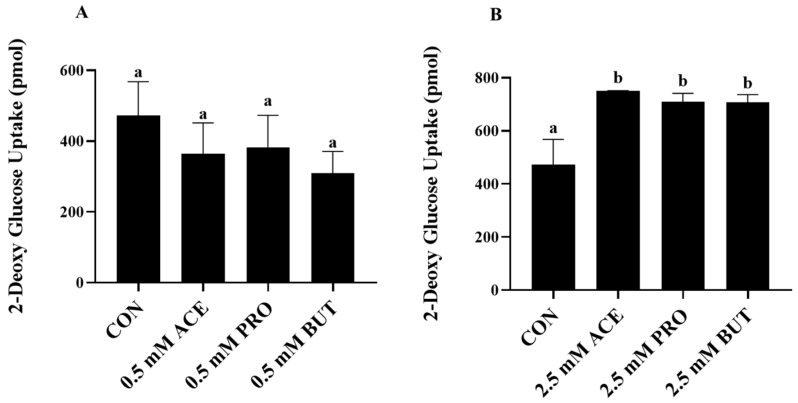
Effect of 0.5 mM and 2.5 mM SCFA + combined LPS and PA stimulation on insulin-stimulated glucose uptake ((**A**) and (**B**), respectively). Values are means ± SEM. Data were analyzed via one-way ANOVA followed by Tukey’s multiple comparison test. Bars not sharing a lower-case letter differ (*p* < 0.05).

Insulin-stimulated glucose uptake was measured in LPS + PA-stimulated L6 myotube cultures to determine if the changes in inflammatory transcription factor activation and inflammatory cytokine and chemokine secretion in response to SCFA treatment were associated with a functional metabolic outcome, as shown in Figure 5. In response to the lower SCFA concentration (0.5 mM), there was no difference in insulin-stimulated glucose uptake between CON and any SCFA treatment group in response to combined LPS + PA stimulation (*p* > 0.05; Figure 5A). Conversely, at the higher 2.5 mM concentration, each of the three SCFA treatments (ACE, PRO, and BUT) increased insulin-stimulated glucose uptake compared to CON (*p* < 0.05; Figure 5B); however, there was no difference in the amount of glucose uptake observed between each individual SCFA, which did not differ from each other.

## 4. Discussion

In an L6 myotube cell culture model designed to recapitulate the critical features associated with obese skeletal muscle, namely via combined stimulation with LPS and PA, we demonstrated the effects of individual SCFAs (ACE, PRO, and BUT) on reducing inflammatory mediator secretion and inflammatory transcription factor activation and increasing insulin-stimulated glucose uptake. The LPS dose utilized in this study mimics the circulating levels of endotoxin observed in obese humans and rodents [44,45,46], whereas the PA dose has been shown to induce insulin resistance in skeletal muscle cells while maintaining cell viability [41,42]. Both LPS and PA have been used as individual stimulation conditions to modulate myocyte inflammatory mediator secretion [40]; however, the combined effects of LPS + PA have not been evaluated together, and when combined, they more accurately mimic the critical features of obese skeletal muscle microenvironments in vivo (namely, inflammation and insulin resistance/impaired glucose homeostasis) [37,38,39]. Thus, this study provides a model for discerning the acute effects of individual SCFAs on local inflammatory mediators (i.e., myokines), which exert autocrine or paracrine effects on myocyte metabolic function when produced locally [37,38]. 

The effects of SCFAs on myocyte function were observed using two different concentrations that have been used previously in cell culture studies utilizing L6 myotubes [28,40,48]. Importantly, these SCFA concentrations reflect the concentration range measured in human blood [18,19,20,21,22,23]. This variability is influenced by the dietary intake of the amount and type(s) of NDC (namely, SCFA precursors, e.g., soluble fibers, resistant starches, oligosaccharides, etc.), the relative fermentability of NDC by the microbiota (i.e., fast versus slow fermentation), the microbiota composition of SCFA-producing microbial species, and intestinal SCFA absorptive capacity [11,12,13,14]. Collectively, this highlights the need to determine the effects of SCFAs at lower and more physiologically relevant concentrations and the amount of each SCFA required to exert a beneficial effect in extra-intestinal tissues. Determining the amount of SCFAs required to modulate extra-intestinal function can allow for more targeted and relevant dietary NDC intake recommendations to be established. However, if higher concentrations of SCFAs are necessary to elicit beneficial extra-intestinal effects that are unattainable through dietary precursor intake and microbial fermentation processes, alternative delivery/supplementary approaches will need to be developed. The concentration of SCFAs used to impact metabolic effects in skeletal muscle in previous studies is variable, ranging between 0.05 and 5 mM [28,30,31,48] or determined on a body weight basis [29,32,36]. In previous studies, a 0.5 mM acetate molar concentration (0.5 mM) induced metabolic improvements in L6 myotubes [28,48] and reduced inflammatory transcription factor activation and subsequent myokine secretion [40]. Therefore, the 0.5 mM SCFA dose serves as a more physiologically relevant SCFA concentration that better reflects the variable circulating concentration levels in vivo [18,19,20,21,22,23]. The higher concentration of 2.5 mM used in this study is central within the range of SCFA molar concentrations used experimentally to demonstrate beneficial effects in vascular smooth muscle cells [48,50,51,52,53,54] and in cardiomyocytes [55]. As important as it is to first determine the individual effects of each SCFA, an important perspective to consider in future studies will be to determine the extra-intestinal effects of SCFAs in combination, thus determining the optimal molar concentrations and ratios of different SCFAs to optimize skeletal muscle function and other extra-intestinal tissues and cell types. 

Previously, to our knowledge, we were the first to demonstrate the effects of individual SCFAs on the L6 myotube’s inflammatory status (specifically, the activation of inflammatory transcription factors and the secretion of inflammatory cytokines and chemokines (i.e., myokines)) in response to separate stimulation with either LPS or PA, which was dependent on their molar concentration and type/carbon number [40]. Thus, each SCFA exerted both unique and some overlapping effects on skeletal muscle cell inflammatory status in response to either LPS or PA alone, wherein butyrate had the most potent non-inflammatory effect and reduced myokine secretion following the pattern of butyrate > propionate > acetate [40]. In the current study, we combined LPS and PA stimulation to more accurately recapitulate the critical features of the obese skeletal muscle cellular microenvironment, which includes LPS-mediated inflammatory signaling and PA-mediated insulin resistance [37,38,39,41,42,43,44,45,46]. In response to combined LPS + PA stimulation at the lower 0.5 mM SCFA concentration, BUT exerted the most potent effect, reducing the secretion of both IL-6 and RANTES, whereas PRO and ACE also reduced RANTES secretion to lesser degrees compared to BUT (Figure 1). Furthermore, at the lower SCFA concentration, only BUT significantly reduced intracellular protein levels of activated (i.e., ratio of phosphorylated–total) inflammatory transcription factors NFκB p65 and STAT3 (Figure 2), thereby highlighting a unique effect of BUT that is not apparent at this concentration in ACE- and PRO-stimulated myotube cultures. In contrast, at the higher (2.5 mM) SCFA concentration, all three SCFAs individually reduced the intracellular protein expression of both NFκB p65 and STAT3 (Figure 4), thereby highlighting how variations in SCFA concentrations (i.e., the high versus the low concentrations tested herein) can dramatically influence the transcriptional capacity for inflammatory mediator production. In this connection, at the higher 2.5 mM SCFA concentration, both BUT and PRO reduced the secretion of IL-6, MCP-1, and RANTES, whereas ACE only reduced RANTES secretion and had no effect on any other myokine assessed (Figure 3), again highlighting the differential effects of different SCFAs. In obesity, the increased secretion of inflammatory mediators, including but not limited to IL-6 and MCP-1, has been shown to contribute to impaired glucose uptake and insulin resistance [37,56,57]. In obesity, there is a cyclic inflammatory process that contributes to sustained low-grade chronic inflammation and the production of inflammatory mediators that contribute to metabolic dysfunction from multiple tissue and cellular sources, including skeletal muscle [37,58,59]. In this connection, increased NFκB signaling has been shown to be a critical driver of skeletal muscle mitochondrial dysfunction, impaired insulin signaling, and reduced glucose uptake [60]. Moreover, the activation of inflammatory transcription factors (such as NFκB and STAT3) drives the production of inflammatory mediators and increases the concentrations of free fatty acids, including palmitic acid, that serve as an inflammatory signal to further induce expression and the secretion of inflammatory cytokines, such as IL-6 and TNFα, via the NFκB and STAT3 pathways [61,62]. Additionally, the elevated circulating LPS levels in obesity serve as additional systemic inflammatory stimuli, signaling through Toll-like receptor 4 (TLR4), leading to the activation of NFκB [63,64] and STAT3 [65], and resulting in the increased production of inflammatory mediators (e.g., TNFα, IL-6, MCP-1, etc.). Collectively, inflammatory chemokines drive the recruitment of immune cell populations into tissues (i.e., adipose tissue, skeletal muscle, liver, etc.) [37,58,59], which serve as additional cellular sources within tissues to further perpetuate inflammatory mediator production/secretion. These inflammatory mediators, such as (but not limited to) TNFα, IL-6, and MCP-1, which are produced by myocytes or obese skeletal muscle, have been shown to contribute to impaired glucose uptake and insulin resistance [37,57,58,59].

The ability of SCFAs to reduce local skeletal muscle-associated inflammation has the potential to also beneficially affect skeletal muscle metabolic function. Therefore, to connect SCFA-mediated changes in the inflammatory status with a functional outcome, insulin-mediated glucose uptake was assessed in LPS + PA-stimulated L6 myotube cultures, wherein all three individual SCFAs increased glucose uptake but only at the 2.5 mM concentration (Figure 5B), whereas there was no effect at the lower 0.5 mM concentration (Figure 5A). Importantly, these results were obtained using physiologically relevant obesity-associated stimulation conditions [37,38,41,42,43,44,45,46] and SCFA concentrations [18,19,20,21,22,23,28,48], whereas supraphysiological SCFA concentrations (20 mM) have been shown to increase glucose uptake in C2C12 myotubes [49]. The combined inflammatory and metabolic outcomes in an obese skeletal muscle cellular microenvironment identified in the current study add to the body of literature in a non-obese context that demonstrates the metabolic effects of SCFA on skeletal muscle function, which includes increased insulin-stimulated glucose uptake and/or improved skeletal muscle glucose tolerance in vivo [28,29,30,31,32,36,48,49], along with individual SCFA-specific effects on lipid metabolism and/or decreasing skeletal muscle lipid accumulation [28,32,33,34,35,48]. Further studies are required to determine the functional implications of reduced skeletal muscle chemokine secretion on immune cell chemotaxis and examine the effects of individual SCFAs on myocyte–immune cell paracrine signaling. 

## 5. Conclusions

Collectively, the results of the current study utilizing combined LPS + PA stimulation and our previous work that utilized separate LPS and PA stimulation conditions [40] demonstrate that different SCFAs are not physiologically equivalent, although they are frequently assumed to be functionally similar. Each SCFA of varying carbon number (acetate, propionate, and butyrate) is in fact a biologically unique molecule that exhibits varying binding affinities for different signaling receptors (including G protein-coupled receptors (GPR)-41, -43, and 109a and the aryl hydrocarbon receptor) and the inhibition of histone deacetylases, as reviewed elsewhere [66,67]. Additionally, the responsiveness of different metabolically active tissues, such as skeletal muscle, to SCFA-mediated signaling will also differ based on the distribution and expression of various SCFA signaling receptors [66]. Thus, the unique effects of individual SCFAs within skeletal muscle are akin to the variability observed with individual SCFAs on intestinal epithelial barrier function [68,69,70,71,72,73,74,75]. Therefore, future studies are required to determine the specific signaling mechanisms utilized by each SCFA to influence both inflammatory mediator secretion and metabolic function and to assess the combined effects of individual SCFAs at physiologically relevant ratios [49]. This mechanistic information in the target tissue (i.e., skeletal muscle) that identifies the concentration of SCFAs required extra-intestinally to improve inflammatory and/or metabolic function will better inform dietary NDC recommendations to promote the sufficient intake of SCFA precursors in order to permit microbial fermentation and SCFA production. This will allow for adequate concentrations of SCFA to be produced intestinally, and via systemic circulation, reach the peripheral tissues to beneficially impact host physiological function and support skeletal muscle function. 

## Data Availability

Data are contained within the article.
